# Competence of Combined Low Dose of Human Chorionic Gonadotropin (HCG) and Clomiphene Citrate (CC) Versus Continued CC during Ovulation Induction in Women with CC-Resistant Polycystic Ovarian Syndrome: A Randomized Controlled Trial

**DOI:** 10.3390/medicina60081300

**Published:** 2024-08-12

**Authors:** Mahmoud Thabet, Mohamed Sayed Abdelhafez, Maged Ragheb Elshamy, Ibrahim A. Albahlol, Emad Fayala, Alaa Wageeh, Ahmed Abdelhamid El-Zayadi, Nagwan Ahmed Bahgat, Shereen M. Mohammed, Alhussein Ahmed Mohamed, Mahmoud Mohamed Awad, Ahmed El-Menayyer, Mohamed El-Sherbiny, Dalia Mahmoud Abdelmonem Elsherbini, Rayan G. Albarakati, Ahmed Baker A. Alshaikh, Fawaz E. Edris, Nayla Jamal Bushaqer, Youstina Georges Makarious Salama, Mahmoud Mohamed Abdel-razik

**Affiliations:** 1 Department of Obstetrics and Gynecology, Faculty of Medicine, Mansoura University, Mansoura 35111, Egypt; mahmoud_thabet@mans.edu.eg (M.T.); m_sayed77@mans.edu.eg (M.S.A.); maged66@mans.edu.eg (M.R.E.); ibrahimelbahlool@yahoo.com (I.A.A.); emadfyala@mans.edu.eg (E.F.); alaawageh@mans.edu.eg (A.W.); ahmedelzayadi@mans.edu.eg (A.A.E.-Z.); nagwanivm1@yahoo.com (N.A.B.); sh_elwakeel@mans.edu.eg (S.M.M.); hossini@mans.edu.eg (A.A.M.); mahmoudmm8506@mans.edu.eg (M.M.A.); ahmed.elmonuer@mans.edu.eg (A.E.-M.); dr_mahmoudabdelrazik@mans.edu.eg (M.M.A.-r.); 2 Department of Obstetrics and Gynecology, College of Medicine, Jouf University, Sakaka 72388, Saudi Arabia; abalshaikh@ju.edu.sa; 3 Department of Basic Medical Sciences, College of Medicine, AlMaarefa University, P.O. Box 71666, Riyadh 11597, Saudi Arabia; msharbini@um.edu.sa; 4 Department of Anatomy, Faculty of Medicine, Mansoura University, Mansoura 35516, Egypt; 5 Department of Clinical Laboratory Sciences, College of Applied Medical Sciences, Jouf University, P.O. Box 2014, Sakaka 72388, Saudi Arabia; 6 Department of Clinical Medical Sciences, College of Medicine, AlMaarefa University, P.O. Box 71666, Riyadh 11597, Saudi Arabia; rbarakati@um.edu.sa; 7 Department of Obstetrics and Gynecology, College of Medicine, Umm AlQura University, Makkah 24382, Saudi Arabia; faedris@uqu.edu.sa; 8 Bahrain Defence Force (BDF) Hospital, Riffa P.O. Box 28743, Bahrain; dr.nayla.j.b@gmail.com (N.J.B.); georges.makaroius@bdfmedical.org (Y.G.M.S.)

**Keywords:** clomiphene citrate, polycystic ovarian syndrome, human chorionic gonadotropin, clomiphene resistance, ovulation

## Abstract

*Background and Objectives*: Polycystic ovarian syndrome (PCOS) is a widespread endocrine disorder affecting 5–18% of females in their childbearing age. The aim of this study is to assess the efficacy of combining a low dosage of human chorionic gonadotropin (HCG) along with clomiphene citrate (CC) for stimulating ovulation in infertile women diagnosed with CC-resistant PCOS. *Materials and Methods*: A randomized controlled trial was carried out on 300 infertile CC-resistant PCOS women. All participants were assigned to two groups: the CC-HCG group and the CC-Placebo group. Subjects in the CC-HCG group were given CC (150 mg/day for 5 days starting on the 2nd day of the cycle) and HCG (200 IU/day SC starting on the 7th day of the cycle). Subjects in the CC-Placebo group were given CC and a placebo. The number of ovarian follicles > 18 mm, cycle cancellation rate, endometrial thickness, ovulation rate, clinical pregnancy rate, and occurrence of early ovarian hyper-stimulation syndrome were all outcome variables in the primary research. *Results*: Data from 138 individuals in the CC-HCG group and 131 participants in the CC-Placebo group were subjected to final analysis. In comparison to the CC-Placebo group, the cycle cancellation rate in the CC-HCG group was considerably lower. The CC-HCG group exhibited a substantial increase in ovarian follicles reaching > 18 mm, endometrial thickness, and ovulation rate. The clinical pregnancy rate was higher in the CC-HCG group (7.2% vs. 2.3%; CC-HCG vs. CC-Placebo). Upon adjusting for BMI and age, the findings of our study revealed that individuals in the CC-HCG group who had serum prolactin levels below 20 (ng/mL), secondary infertility, infertility duration less than 4 years, baseline LH/FSH ratios below 1.5, and serum AMH levels more than 4 (ng/mL) had a higher likelihood of achieving pregnancy. In the CC-Placebo group, there was a greater prediction of clinical pregnancy for those with serum AMH (<4), primary infertility, serum prolactin ≤ 20 (ng/mL), baseline LH/FSH < 1.5, and infertility duration < 4 years. *Conclusions*: The use of a small dose of HCG along with CC appeared to be an effective treatment in reducing cycle cancelation, improving the clinical pregnancy rate and ovulation rate in CC-resistant PCOS patients. The trial was registered with Clinical Trials.gov, identifier NCT02436226

## 1. Introduction

Polycystic ovarian syndrome (PCOS) is a widespread endocrine ailment that affects 5–18% of women in their childbearing age and is a frequent reason for anovulatory infertility being verified in almost 75% of these cases [1]. Along with chronic anovulation, women with PCOS are commonly presented with hyperandrogenism or clinical hyperandrogenism, namely, acne, alopecia, type II diabetes mellitus, obesity, insulin resistance, and hypertension [2,3,4]. Healthcare practitioners and women with PCOS should be apprised that women with PCOS are more likely to develop type 2 diabetes, impaired glucose tolerance, and impaired fasting glucose, regardless of age or BMI. Insulin resistance is a pathophysiological component in PCOS; however, insulin assays have little clinical value and are not recommended in conventional therapy [5]. Since the Rotterdam criteria do not address this component, the medical community has lately begun to see them as deceptive diagnostic tools, raising questions about whether the name PCOS is appropriate to represent all of the reported clinical signs [6]. For individuals with PCOS, ovulation induction by CC is still the major treatment option, despite the availability of other methods that are easy and cost-effective compared to others [7,8,9]. Chera-Aree et al. [10] and Fu and Kuang [11] stated that letrozole had been considered as another first-line treatment for ovulation induction in PCOS. Chera-Aree et al. [10] proved that the ovulation rates attained with a combination of CC and letrozole were not significantly different from those attained with CC 50 mg alone in one cycle. Their findings indicated that CC is the key driver of ovulation and that CC alone may be a commercially feasible ovulation induction treatment for both PCOS patients who are not resistant to CC and overall anovulatory women. The ability of CC to induce ovulation comes from its indirect role in releasing follicle-stimulating hormone (FSH) from the anterior pituitary gland in quantities enough to reset the sequence of events that culminate in ovulation. It adheres to estrogen receptors in the hypothalamus, inhibiting estrogen hormone binding to these receptors. The hypothalamus recognizes a lack of estrogen binding at the receptors and secretes gonadotropin-releasing hormone (GRH). GRH stimulates the pituitary gland to generate more follicle-stimulating hormone (FSH) and luteinizing hormone (LH) [10,12,13]. Clomiphene citrate in PCOS is commonly recommended for 5 days from the 2nd or 3rd day of menstruation; the initial dose of 50 mg/day was increased reaching 250 mg/day, despite the most effective dosage proving to be 100–150 mg/day [14]. The treatment may be extended for 6 to 9 cycles with a proven cumulative success rate reaching up to 70–75% [14,15]. Resistance to CC occurs in 15–40% of patients, and is diagnosed after administering 150 mg/day of CC for 5 days for at least three anovulatory cycles [16]. This failure in most cases is virtually unpredicted and might be a non-explainable event, but it is proven to be more common in obese patients, where insulin resistance, hyperandrogenic state, and associated genetic predispositions may all have a role [17].

As there is no widespread consensus on the conventional therapy of CC resistance, many ways were tried, aiming to increase ovulation and subsequent pregnancy rates. Some earlier studies advised the management to shift after proving resistance to gonadotropins and laparoscopic ovarian drilling [18]. However, other researchers established the benefit of letrozole treatment, demonstrating a substantial rise in the probability of ovulation in CC-resistant PCOS subjects as compared to ovarian drilling [19]. Prior meta-analytic studies have shown notable variability across randomized controlled trials examining the ovulation rates attained with letrozole and CC in individuals with PCOS. However, letrozole demonstrated a considerable increase in the ovulation rate when compared to CC [9,20]. On the contrary, a meta-analysis of trials on unexplained infertility in women that compared the effectiveness of letrozole with that of CC showed no substantial disparities in clinical findings between the two groups [10,21,22]. A previous study recommended the incorporation of N-acetyl cysteine, metformin, and glucocorticoids with the expectation of achieving an improved ovarian response [23]. Recently, some have tried the effectiveness of extended or intermittent CC on ovulation and pregnancy rates in PCOS patients with CC resistance with convenient results [24,25]. Human chorionic gonadotropin (HCG) is a hormone secreted by the placenta. It is first created by syncytiotrophoblast cells in the implanting conceptus during the second week of pregnancy. HCG supports the ovarian corpus luteum, which, in turn, sustains the endometrial lining and ensures the continuation of the pregnancy [26]. HCG was chosen because of its low cost, ability to function as a surrogate for LH, and its ability to occupy LH receptors for more than 24 h, which allows stable stimulation of the LH receptors [27]. Low-dose HCG can be administered to patients being stimulated for assisted reproduction to complete folliculogenesis. Prior research indicated that low-dose HCG was more successful than usual regimens for establishing pregnancy. Filicori et al. demonstrated that in the last phases of ovarian stimulation, folliculogenesis may be promoted by administering low-dose HCG (200 IU daily for 7 days) [28]. This strategy of ovarian stimulation is both cost-effective and efficient and represents an effective alternative to gonadotropin therapy in subjects with CC-resistant anovulation [29]. The goal of this study is to appraise the efficacy of adding a small dose of HCG in PCOS women with proven CC resistance.

## 2. Materials and Methods

### 2.1. Study Design and Population

This research was a prospective, randomized, controlled, parallel-group trial that was performed from March 2016 to January 2023 at the Obstetrics and Gynecology Department of the Fertility Care Unit at Mansoura University Hospitals in Mansoura, Egypt. The protocol went through review and approval by the Institutional Research Board of Mansoura Faculty of Medicine (Code No. R/15.08.23). Additionally, the trial was registered with ClinicalTrials.gov, identifier NCT02436226. Consequently, the study was conducted in compliance with the ethical standards outlined in the 1964 Declaration of Helsinki and its subsequent amendments.

The research included women with (PCOS) who were identified using Rotterdam Consensus 2004 [30] criteria. Diagnosis was based on the existence of at least two out of the following criteria: (1) anovulation or oligo-ovulation; (2) indications of excessive androgen levels either by laboratory tests or clinical manifestations; and (3) Transvaginal sonography (TVS) evaluation for polycystic ovarian morphology. In women, clinical symptoms of high androgen levels involve hirsutism, acne, and female pattern hair loss. The grade of hair growth is commonly evaluated using the Modified Ferriman–Gallwey (MFG) scoring method, in which terminal hair growth is rated on a scale from 0 to 4 at nine separate anatomic locations, and scores are measured. Different MFG score limits have been suggested to diagnose hyperandrogenism, which range from ≥3 to ≥8 [31]. Biochemical hyperandrogenism was determined by higher total or free testosterone levels as evaluated by high-quality tests. Free testosterone, free androgen index, or bioavailable testosterone may be used in the diagnosis of PCOS as markers of biochemical hyperandrogenism [31], All the possible participating women were interviewed, provided with comprehensive information on the research’s procedure, and then advised on their enrollment in the study. The inclusion criterion was infertile PCOS having clomiphene resistance, recognized as ovulation failure after taking a daily dose of 150 mg of (CC) for 5 consecutive days each menstrual cycle, for a minimum of at least 3 consecutive cycles [32].

The exclusion criteria were (1) body mass index (BMI) is > 30 or < 18.5 kg/m^2^); (2) age is > 35 or < 18 years; (3) individuals with any other infertility attribute apart from anovulatory PCOS; (4) individuals with a history of ovarian surgery or unilateral oophorectomy; (5) previously subjected to pelvic irradiation or cytotoxic drugs; (6) use of hormonal or oral hypoglycemic management either currently or within 3 months prior; or (7) subjects with abnormal thyroid function, with hyperprolactinemia, with primary anovulation (FSH > 10), and subjects with uncontrolled diabetes.

### 2.2. Initial Evaluation

A complete history was taken from each participant, including personal history, menstrual history, obstetric history (gravidity and parity to determine the type and duration of infertility), medical history, and surgical history. Following a general examination, the patient’s weight in kilograms (kg) and height in centimeters (cm) were noted, and their BMI was computed. Every subject had their baseline levels of prolactin, thyroid stimulating hormone (TSH), anti-Müllerian hormone (AMH), LH, and FSH measured in the blood.

### 2.3. Randomization

Participants in the research were randomized into two groups on the 1st day of the menstrual cycle: the CC-HCG group and the CC-Placebo group. The randomization process was performed by a nurse using opaque, sealed, unmarked envelopes with computer-generated random numbers inside. The randomization was balanced (allocation ratio to each group was 1:1) and simple, and the study was a double-blinded study (i.e., the participants, investigators, and caregivers were not aware of group allocation).

### 2.4. Ovarian Stimulation Protocol

In the CC-HCG group, women were given 150 mg/day of CC initiated on day 2 of the cycle and 200 IU/day SC of HCG (Choriomon^®^, HCG 5000 IU, IM injection, IBSA (Institut Biochimique SA), Lugano, Switzerland) starting on day 7 of the cycle after induction of ovulation to complete folliculogenesis for approximately 7 days for a maximum of 10 days (from 7th till day 16th of the cycle) with a maximum HCG dose of 2000 IU [28,29]. In the CC-Placebo group, women were given distilled water (0.12 mL SC) on day 7 of the cycle as a placebo and CC (150 mg/day) for five days in succession starting on day two of the cycle. On the tenth day of the stimulation cycle, the same sonographer began monitoring the development of the follicles using TVS scanning, or folliculometry, and repeated the procedure every two to three days. Final oocyte maturation was induced when at least one follicle measured ≥ 18 mm in diameter. If the follicle did not reach more than 12 mm by day 16th, the cycle was canceled and assumed to be anovulatory. Human chorionic gonadotropin (10,000 IU IM) was given when the mean diameter of the lead follicle was 18 mm or larger. Timed intercourse was recommended on the day of stimulating ovulation and the following day. Ovarian stimulation was administered to each woman for a maximum of three consecutive cycles except if she ovulated in the 1st or 2nd cycle.

### 2.5. Ovulation Documentation

After ovulation triggering, all participants were followed up 3 days using TVS scanning for detection of signs of ovulation: (1) vanishing of the follicle or sudden reduction in its size; (2) presence of free fluid in the pelvis or Douglas pouch; (3) increased echogenicity of the follicle, indicating formation of corpus luteum; and (4) substitution of the triple line endometrial shape by the hyperechoic, homogenous luteinized endometrium.

### 2.6. Luteal Phase Support

Progesterone vaginal suppositories (Prontogest^®^, Progesterone 200 mg, vaginal or rectal pessaries, Marcyrl Pharmaceutical Industries, Cairo, Egypt) were given to all participants every 12 h, starting the day after ovulation was triggered.

### 2.7. Pregnancy Documentation

A quantitative serum beta-HCG (-HCG) level was evaluated using immunoassay in women who had missed their menstrual period for one week, and a serum HCG level > 50 mIU/mL was deemed indicative of biochemical pregnancy. TVS scanning was performed on women with biochemical pregnancy 6–8 weeks after the first day of their last menstrual cycle to confirm a clinical intrauterine pregnancy, which is described as the existence of at least one intrauterine gestational sac with an embryonic pole and heart pulse on TVS scanning at 6–8 weeks of gestational period.

### 2.8. Outcomes

The primary outcome was the ovulation rate, estimated by dividing the number of ovulatory cycles by the number of stimulation cycles. The secondary outcomes were the following: cycle cancelation rate (estimated by dividing the number of canceled cycles by the number of stimulation cycles); number of ovarian follicles ≥ 18 mm on the day of HCG administration; clinical pregnancy rate (estimated by dividing the number of cycles in which clinical pregnancy had occurred by the number of stimulation cycles); endometrial thickness (measured on day of HCG administration (in women received triggering with timed intercourse) or on day of last TVS scan during folliculometry (in women with cycle cancelation)); and occurrence of early ovarian hyper-stimulation syndrome (OHSS).

### 2.9. Statistical Analysis

Data were collected, tabulated, and Statistical Package for the Social Sciences (SPSS version 22.0. IBM Corp., Armonk, NY, USA) was used for statistical analysis. The chi-square test was performed to examine the relationship of categorical parameters across groups. In parametric data, Student’s *t*-test was employed to compare means of quantitative measures. The odds ratio (with 95% confidence interval) and a *p*-value of 0.05 or less were used to analyze the predictive variables for clinical pregnancy and ovulation. Graph Pad software version 9 was used to plot the predictive variables.

## 3. Results

### 3.1. Baseline Data

**Figure 1:**
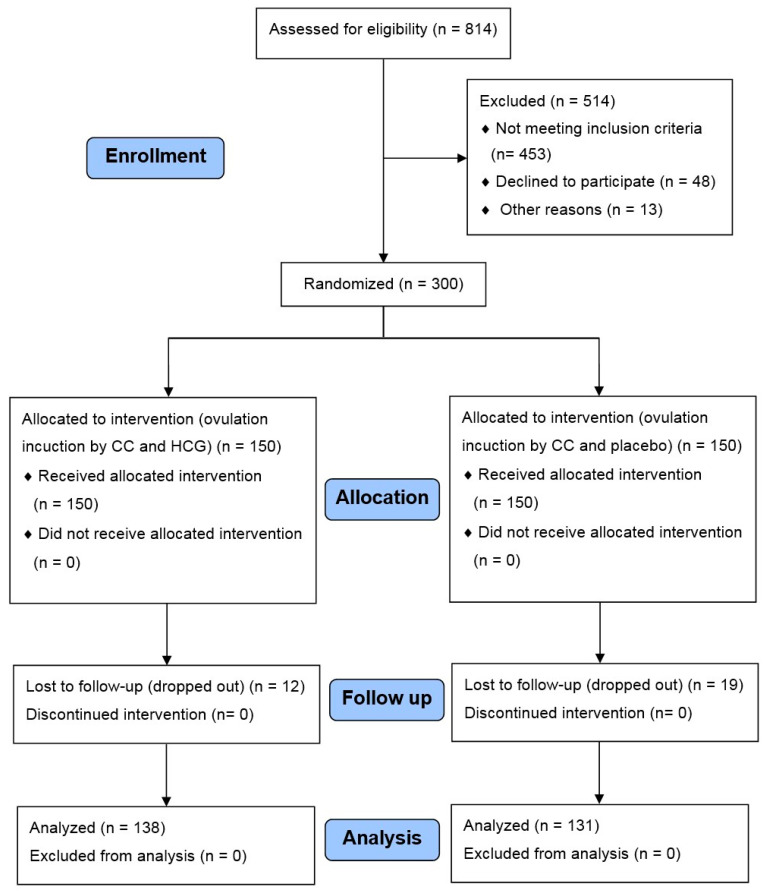
Study flow diagram.

A total of 814 patients with clomiphene-resistant PCOS were included in the trial; 514 patients were excluded owing to failure to fulfill the inclusion criteria (453), refusal to participate (48), or other reasons (difficult follow-up due to difficult transportation or with endometrial pathology proved by transvaginal ultrasound (TVS)) (13) (Figure 1). The remaining patients were equally assigned into two groups: the CC-HCG group and the CC-Placebo group, but 12 patients from the first group and 19 from the second were lost to follow-up. Therefore, 138 cases representing the CC-HCG group and 131 representing the CC-Placebo group were subjected to statistical analysis.

### 3.2. Baseline Characteristics

Baseline data are summarized in Table 1; there were no significant differences between study groups in patients’ age, body mass index, infertility type, or duration, serum AMH, FSH, LH, baseline LH/FSH, prolactin, or TSH (mIU/mL), and *p* > 0.05.), the mean value of baseline LH/FSH is < 1.5.

### 3.3. Outcomes

Table 2 illustrates the outcomes. The mean estimated number of follicles ≥ 18 mm was significantly higher in the CC-HCG group. Cycle cancelation [100 (72.5%) vs. 122 (93.1%)], endometrial thickness [8.07 ± 1.67 vs. 7.24 ± 1.06], rate of ovulation [37 (26.8%) vs. 8 (6.1%)] were significantly higher in CC-HCG group than CC-Placebo group, *p* < 0.05. On the other hand, no significant difference regarding clinical pregnancy and OHSS in both groups 10 (7.2%), vs. 3 (2.3%) and 2 (1.4%) vs. 1 (0.8%), respectively.

### 3.4. Univariate Analysis for Predictors of Clinical Pregnancy

**Figure 2:**
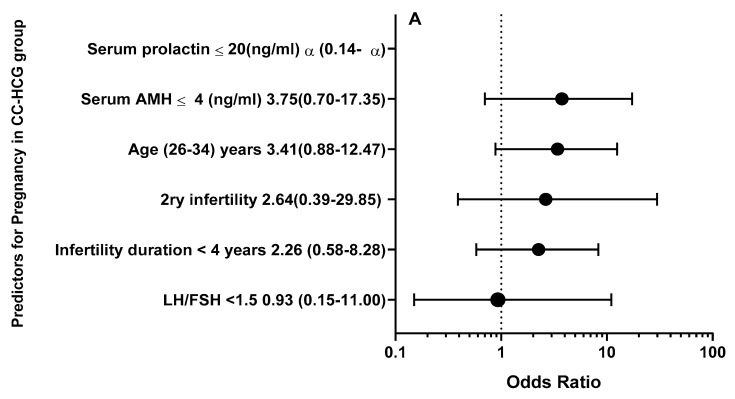

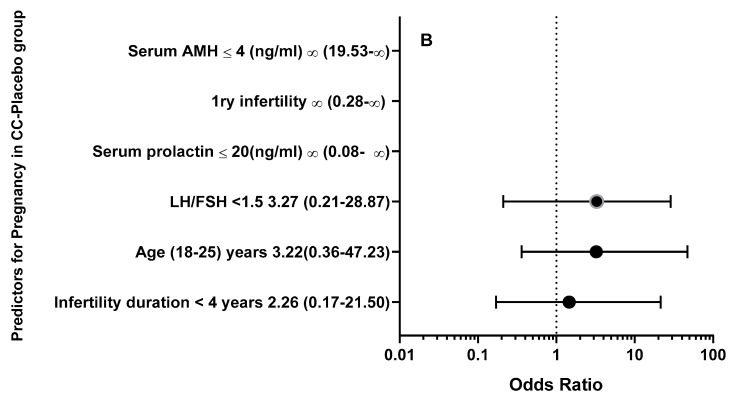
Forest plot for odds ratio and 95% confidence interval for clinical pregnancy predicting factors in (**A**) CC-HCG group and (**B**) CC-Placebo group.

After adjusting for BMI and age, logistic regression analyses showed that serum prolactin ≤ 20 (ng/mL) (odds ratio [OR], ∞ [95% CI, 0.14–∞], *p* = 0.99), serum AMH ≤ 4 (ng/mL) (OR, 3.75 [95% CI, 0.70–17.35], *p* < 0.70), secondary infertility (OR, 2.64 [95% CI, 0.39–29.85], *p* = 0.70), infertility duration < 4 years (OR, 2.26 [95% CI, 0.58–8.28], *p* = 0.33), and baseline LH/FSH (OR, 0.93 [95% CI, 0.15–11.00], *p* = 0.95) were correlated with a higher prediction of clinical pregnancy in CC-HCG group. On the other hand, serum AMH ≤ 4 (OR, ∞ [95% CI, 19.53–∞], *p* < 0.001), primary infertility (OR, ∞ [95% CI, 0.28–∞] *p* = 0.99), serum prolactin ≤ 20 (ng/mL) (OR, ∞ [95% CI, 0.08–∞], *p* = 0.99), baseline LH/FSH (OR, 3.27 [95% CI, 0.21–28.87], *p* = 0.32), and infertility duration < 4 years (OR, 1.46 [95% CI, 0.17–21.50], *p* = 0.99) were correlated with a higher prediction of clinical pregnancy in CC-Placebo group (Table 3, Figure 2).

### 3.5. Univariate Analysis for Predictors of Ovulation

**Figure 3:**
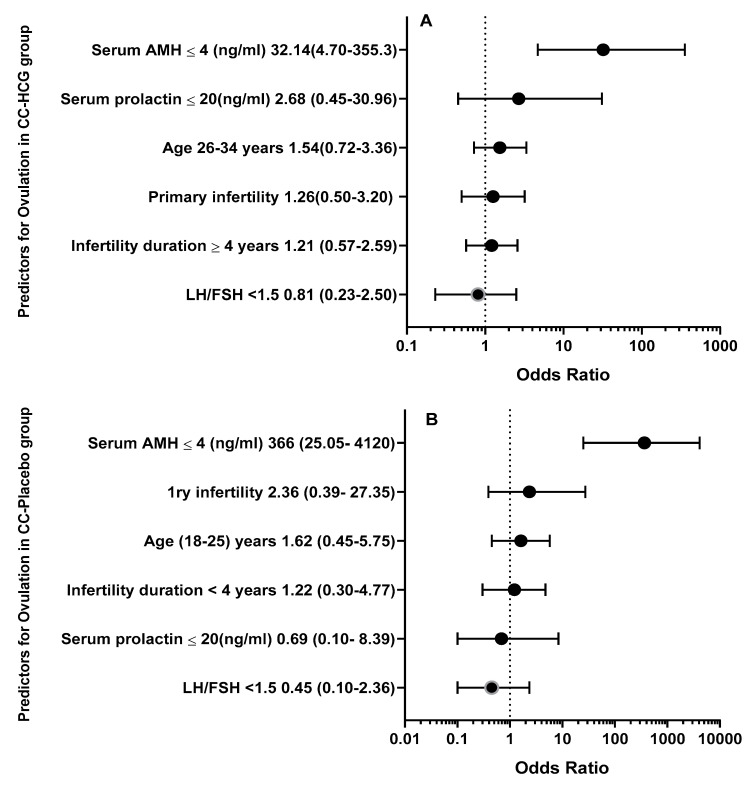
Forest plot for odds ratio and 95% confidence interval for ovulation predicting factors in (**A**) CC-HCG group and (**B**) CC-Placebo group.

Logistic regression analyses showed that serum AMH ≤ 4 (ng/mL) (OR, 32.14 [95% CI, 4.70–355.3], *p* < 0.001), serum prolactin ≤ 20 (ng/mL) (OR, 2.68 [95% CI, 0.45–30.96], *p* = 0.68), primary infertility (OR, 1.26 [95% CI, 0.50–3.20], *p* = 0.82), infertility duration ≥ 4 years (OR, 1.21 [95% CI, 0.57–2.59], *p* = 0.70), and baseline LH/FSH (OR, 0.81 [95% CI, 0.23–2.50], *p* = 0.74), were correlated with a higher prediction of ovulation in CC-HCG group. On the other hand, basal serum AMH ≤ 4 (OR, 366 [95% CI, 25.05–4120], *p*< 0.001), primary infertility (OR, 2.36 [95% CI, 0.39–27.35] *p* = 0.68), infertility duration < 4 years (OR, 1.22 [95% CI, 0.30–4.77], *p* = 0.99), serum prolactin ≤ 20 (ng/mL) (OR, 0.69 [95% CI, 0.10–8.39], *p* = 0.55), and baseline LH/FSH (OR, 0.45 [95% CI, 0.10–2.36], *p* = 0.34) were correlated with a higher prediction of ovulation in CC- Placebo group (Table 4, Figure 3).

## 4. Discussion

The main findings of the study confirmed that the addition of small dose HCG could improve the ovulation rate, and to a lesser extent, the clinical pregnancy rate. This is indicated by the determination of the number of follicles reaching or exceeding 18 mm in diameter, number of canceled cycles, and endometrial thickness.

It has been reported that clomiphene citrate, which is regarded as a first-line drug for this purpose with an anticipated resistance rate of 15%, is an essential treatment option for inducing ovulation in PCOS [32,33]. Furthermore, many options were tried to overcome this resistance, but the most frequently used are gonadotropin, aromatase inhibitors, and laparoscopic ovarian drilling [34,35,36]

The value of adding low-dose HCG (100–200 IU) or LH during ovulation induction for normal ovulatory patients or in a controlled ovarian surge as a terminal stage of follicular maturation has previously been proven and universally documented by many authors [37,38,39,40,41,42]. Based on these scientific facts, the authors of this study can state that the results and findings of this work are in accordance with the findings published by the previously mentioned authors.

The improved ovulation rate in CC-resistant PCOS patients, with the addition of a small dose of HCG, was already proven by Branigan and Estes [27]. Additionally, it was observed that PCOS patients undergoing ovulation induction with the progesterone protocol in ICSI cycles experienced a marginal increase in preovulatory follicles and an improvement in the clinical pregnancy rate when a low-dose HCG was administered during the follicular phase [43]. It is believed that HCG may be mediating relevant reproductive processes. Low-dose HCG could also be utilized to imitate LH activities on growing follicles in a more steady and sustained way, allowing folliculogenesis to progress. The stimulatory action of low-dose hCG on follicular maturation is probably due to its interaction with LH receptors expressed by the granulosa cells of larger follicles and the enhanced activation of the granulose cell aromatase enzyme system [44]. This method could mimic hormone dynamics during the normal follicular stage, potentially lowering the likelihood of issues like ovarian hyper-stimulation syndrome [29]. A previous study found that low-dose HCG was much more effective than a conventional regimen at achieving pregnancies. This approach to ovarian stimulation represents an effective and economic alternative [27]. Successful pregnancy depends on a complex interplay between the immune and the endocrine systems to tightly regulate immune responses at the fetal–maternal interface [45]. The pregnancy hormone HCG has been described to have immunoregulatory properties supporting the implantation process of the fetus in the maternal endometrium [46]. In our study, although the ovulation rate was higher in the CC-HCG group compared to the other arm of the study, the pregnancy rate was not significantly increased. This might be attributed to, according to the authors’ thinking, the increased synthesis of ovarian androgen by theca cells in response to HCG stimulation. Similar results were documented by Yilmaz et al. [47] who noticed an increase in ovulation and clinical pregnancy rates in the CC-HCG group but they reported that the difference was insignificant as compared with the CC-alone group. Friis Wang et al. [42] and Kristensen et al. [48] reported that there was a significant increase in the number of small antral follicles after rLH pretreatment. The Inhibin B concentrations were significantly lower on stimulation day 1 after HCG priming, and significantly reduced intrafollicular Inhibin B-level in small antral follicles from women with PCOS as compared to normal women.

It is well known that polycystic ovarian syndrome patients are at higher risk for OHSS due to high follicular pool and increased antral follicle count [40]. Our results showed no significant difference between the studied groups regarding this complication. This finding agrees with other investigators [49,50] and it may be attributed to the lower production of vascular endothelial growth factor and vasopressin by granulosa cells in response to a low dose of HCG, therefore decreasing vascular permeability even when there has been a high level of FSH-stimulated follicle development, a fact which has also previously been explained by other authors [50]. In addition, the small number of patients suffering from hyper-stimulation may be due to a deficient FSH receptor on the follicles, as using small doses of HCG in CC-resistant PCOS patients helps to avoid the administration of high doses of FSH to overcome resistance during ovulation induction. Consequently, the small and intermediate follicles will undergo atresia, as they are deficient in FSH receptors, reducing the incidence of OHSS.

After adjusting for BMI and age, our results showed that serum prolactin ≤ 20 (ng/mL), serum AMH ≤ 4, secondary infertility, infertility duration < 4 years, and baseline LH/FSH < 1.5 were correlated with a higher prediction of clinical pregnancy in the CC-HCG group. While serum AMH ≤ 4, primary infertility, serum prolactin ≤ 20 (ng/mL), baseline LH/FSH < 1.5, and infertility duration < 4 years were correlated with a higher prediction of clinical pregnancy in the CC-Placebo group.

Hassan [51] conducted a study on females with PCOD stimulated by the GnRH (gonadotropin-releasing hormone) antagonist protocol and gonadotropins for approximately 7–14 days. They observed that the pregnancy rate was lower in the females who had hyperprolactinemia. Kamel et al. [52] also reported that hyperprolactinemia decreased cleavage and pregnancy rates.

Our study revealed a non-significant difference in baseline LH/FSH between both groups but it was higher in the CC-Placebo group. This could be attributed to increased BMI and AMH levels in that group. The LH/FSH ratio was significantly associated with age, BMI, and AMH as anovulatory women had a significantly higher LH/FSH ratio than ovulatory women, especially in women with young age, high BMI, and AMH [53]. A greater baseline LH/FSH ratio has been demonstrated to impede human reproduction. Our study revealed that an LH/FSH ratio of less than 1.5 is one of the predictors of ovulation in PCOS patients, which was consistent with a previous study by Xia et al. [53] who reported that an elevated baseline LH/FSH ratio in women with PCOS was associated with poor ovulatory response. Intrauterine hyperandrogen exposure is suggested to be a crucial element in the reprogramming of several genes that contribute to the development of PCOS. This condition affects the hypothalamus–pituitary axis and disrupts metabolism, leading to increased LH production throughout puberty and adolescence [54]. Patients with PCOS have a drop in FSH, which leads to an increase in the ratio of LH/ FSH. This, in turn, results in an elevation in androgen synthesis from theca cells in the ovaries, ultimately leading to excessive production of androgens. Moreover, this condition would hinder the growth of follicles and result in the persistent absence of ovulation. The presence of several small antral follicles results in the development of polycystic ovarian morphology [55,56,57]. PCOS individuals with elevated LH/FSH ratios had a greater likelihood of achieving pregnancy while undergoing treatment with GnRH-agonist protocols as opposed to GnRH-antagonist treatments [58].

Women with PCOS have elevated synthesis of AMH from ovarian granulosa cells in comparison to control subjects. Overactive AMH in granulosa cells reduces the production of the aromatase enzyme, which hinders the development of follicles and prevents ovulation, leading to follicular arrest. As the quantity of follicles that have been apprehended rises, the levels of AMH also rise more [59]. AMH has recently been regarded as a diagnostic or predictive indicator of PCOS [60,61]. While several researchers have shown an inverse correlation between the concentration of anti-Müllerian hormone (AMH) and the incidence of pregnancy, other investigations have discovered a favorable association between AMH concentration, embryo quality, and clinical pregnancy rates [62]. Vagios et al. [63] reported that although the chance of responding to CC was great at low AMH levels, this probability declined as AMH increased. They may postulate that the impact of CC on the endogenous route of gonadotropin production is insufficient to counteract the suppressive impact of AMH when blood levels exceed a specific threshold. The influence of AMH on the outcomes of gonadotropin-induced ovulation is poorly understood. Di Paola et al. [64] proposed that PCOS women with significantly raised blood AMH levels would have a decreased response to ovulation induction with gonadotropins and advocated a greater dose, which might eventually lead to an over-response. According to Vagios et al. [63], using gonadotropins as the first choice for ovulation induction (OI) may be more effective, especially when taking a conservative approach to stimulation. Alternatively, increasing the initial dosage of oral agents could be advantageous for specific subgroups of PCOS patients with elevated serum AMH levels and a higher likelihood of resistance to orally administered OI agents. In their study, they found that almost all cycles using gonadotropins resulted in ovulation, regardless of the individual’s AMH levels. However, cycles with AMH levels over the 75th or 90th percentile (14.0 or 19.0 ng/mL, respectively) of the population using oral ovulation induction drugs exhibited significantly lower response rates.

Regarding infertility duration as a predictor of pregnancy in subjects with PCOS, similar results were reported by van Wely et al. [65] and Si et al. [66] who observed that women with infertility duration of < 2 years were found to be more likely to achieve an ongoing pregnancy following ovulation induction with recombinant FSH in women with polycystic ovary syndrome, but this association was not statistically significant. Patients with shorter infertility duration had a higher chance of live birth, and duration was a significant predictor of live birth [66,67,68]. This could be attributed that some people with PCOS have prolonged periods of infertility because of anovulation or oligo-ovulation, which may result in lost opportunities to conceive. According to McDonnell and Hart [69], PCOS is the most prevalent cause of anovulatory infertility. However, previous research conducted by Guan et al. [70] found no significant difference between the pregnancy and non-pregnancy groups regarding the infertility duration after stimulation of ovulation in subjects with PCOS.

In our study, the clinical pregnancy rate was higher in subjects with secondary infertility in the CC-HCG group. Similar results were reported by Guan et al. [70] who hypothesized that women who have had previous pregnancies may encounter irregular menstruation, ovulation abnormalities, and polycystic ovarian syndrome as a result of weight increase, subsequently leading to secondary infertility. Oocyte immaturity was the cause of unsuccessful fertilization in these patients. Individuals diagnosed with secondary infertility are more likely to achieve a fruitful pregnancy subsequent to ovarian issue resolution, in contrast to patients who experience primary infertility.

Our study also has limitations. Being a single-center study rather than a multi-center one means that it was less informative, with a more limited number of participants, especially compared to one with a long study period. Another limitation of our study is that we did not try alternative HCG dosing regimens. This could be explained by the fact that, according to the authors’ opinion, the occurrence of the COVID-19 pandemic during the study period caused the dropout of many cases and created fear in patients to attend the fertility care unit to manage their infertility during this difficult and stressful period.

## 5. Conclusions

Adding HCG to CC in CC-resistant PCOS cases could improve follicular growth, the clinical pregnancy rate, and the ovulation rate without increasing the risk of OHSS. This approach has the advantage of low cost and could be used before moving to gonadotropin therapy.

## Figures and Tables

**Table 1:** Demographic, clinical, and hormonal characteristics between both groups.

Variables	CC-HCG Group (*n* = 138)	CC-Placebo Group (*n* = 131)	*p*-Value
Age (years)	26.48 ± 3.73	27.19 ± 3.97	0.163
BMI (Kg/m^2^)	25.88± 2.01	25.95 ± 1.96	0.587
Infertility type Primary Secondary	108 (78.3%) 30 (21.7%)	99 (75.6%) 31 (24.4%)	0.601
Infertility duration (years)	3.53 ± 1.08	3.34 ± 1.06	0.155
Serum AMH (ng/mL)	7.85 ± 2.72	7.98 ± 1.96	0.545
Baseline serum FSH (mIU/mL)	5.50 ± 1.03	5.51 ± 1.20	0.419
Baseline serum LH (mIU/mL)	6.16 ± 1.23	6.12 ± 1.50	0.736
Baseline LH/FSH	1.14 ± 0.23	1.16 ± 0.39	0.541
Serum prolactin (ng/mL)	13.66 ± 3.95	14.04 ± 4.07	0.372
Serum TSH (mIU/mL)	1.97 ± 0.48	1.95 ± 0.48	0.717

Data were expressed as mean (SD), *p*-value was set as significant if <0.05. Abbreviations: BMI; body mass index, AMH; anti-Müllerian hormone, FSH; follicle-stimulating hormone, LH; luteinizing hormone, TSH; thyroid stimulating hormone.

**Table 2:** Outcomes of stimulation protocol among both groups.

Variables	CC-HCG Group (*n* = 138)	CC-Placebo Group (*n* = 131)	*p*-Value
Number of follicles ≥ 18 mm Median (min–max)	0 (0–5)	0 (0–3)	<0.001 *
Cycle cancelation	100 (72.5%)	122 (93.1%)	<0.001 *
Endometrial thickness (mm) Mean ± SD	8.07 ± 1.67	7.24 ± 1.06	<0.001 *
Ovulation	37 (26.8%)	8 (6.1%)	<0.001 *
Clinical pregnancy	10 (7.3%)	3 (2.3%)	0.086
OHSS	2 (1.4%)	1 (0.8%)	1.000

Data are expressed as median (min–max), mean (SD), and frequency (percentage). * *p*-value < 0.05 was considered significant. Abbreviations: OHSS; ovarian hyper-stimulation syndrome.

**Table 3:** Predicting factors for clinical pregnancy in both groups.

Predictors	CC-HCG Group (*n* = 138)			CC-Placebo Group (*n* = 131)		
	Clinical Pregnancy *n* (%)	Odds Ratio (95% CI)	*p*-Value Fisher’s Exact Test	Clinical Pregnancy *n* (%)	Odds Ratio (95% CI)	*p*-Value Fisher’s Exact Test
Infertility type						
-Primary infertility	1 (0.7%)	0.38 (0.03–2.56)	0.70 (ns)	3 (2.3%)	∞ (0.28–∞)	0.99 (ns)
-Secondary infertility	9 (6.5%)	2.64 (0.39–29.85)		0 (0.0%)	0.00 (0.00–2.58)	
Infertility duration (years)						
<4	7 (5.1%)	2.26 (0.58–8.28)	0.33 (ns)	2 (1.5%)	1.46 (0.17–21.50)	0.99 (ns)
≥4	3 (2.2%)	0.44 (0.12–1.71)		1 (0.8%)	0.69 (0.05–6.03)	
Baseline LH/FSH						
<1.5	9 (6.5%)	0.93 (0.15–11.00)	0.95 (ns)	2 (1.5%)	3.27 (0.21–28.87)	0.32 (ns)
≥1.5	1 (0.7%)	1.07 (0.09–6.54)		1 (0.8%)	0.31 (0.03–4.68)	
Serum prolactin (ng/mL)						
≤20	10 (7.3%)	∞ (0.14–∞)	0.99 (ns)	3 (2.3%)	∞ (0.08–∞)	0.99 (ns)
>20	0 (0.0%)	0.00 (0.00–6.98)		0 (0.0%)	0.00 (0.00–11.13)	
Serum AMH (ng/mL)						
≤4	2 (1.5%)	3.75 (0.70–17.35)	0.70 (ns)	3 (2.3%)	∞ (19.53–∞)	<0.001 *
>4	8 (5.8%)	0.27 (0.06–1.43)		0 (0.0%)	0.00 (0.00–0.05)	

* p-value < 0.05 was considered significant. ns = non-significant. Abbreviations: AMH; anti-Müllerian hormone, FSH; follicle-stimulating hormone, LH; luteinizing hormone, ∞; infinity.

**Table 4:** Predicting factors for ovulation in both groups.

Predictors	CC-HCG Group (*n* = 138)			CC-Placebo Group (*n* = 131)		
	Ovulation n (%)	Odds Ratio (95% CI)	*p*-Value Fisher’s Exact Test	Ovulation n (%)	Odds Ratio (95% CI)	*p*-Value Fisher’s Exact Test
Infertility type						
Primary infertility	30 (21.7%)	1.26 (0.50–3.20)	0.82 (ns)	7 (5.3%)	2.36 (0.39–27.35)	0.68 (ns)
Secondary infertility	7 (5.1%)	0.79 (0.31–2.00)		1 (0.8%)	0.42 (0.04–2.55)	
Infertility duration (years)						
<4	18 (13.1%)	0.82 (0.39–1.74)	0.70 (ns)	5 (3.9%)	1.22 (0.30–4.77)	0.99 (ns)
≥4	19 (13.7%)	1.21 (0.57–2.59)		3 (2.3%)	0.82 (0.21–3.31)	
Baseline LH/FSH						
<1.5	33 (23.9%)	0.81 (0.23–2.50)	0.74 (ns)	6 (4.6%)	0.45 (0.10–2.36)	0.34 (ns)
≥1.5	4 (2.9%)	1.24 (0.40–4.40)		2 (1.5%)	2.23 (0.42–10.15)	
Serum prolactin (ng/mL)						
≤20	36 (26.1%)	2.68 (0.45–30.96)	0.68 (ns)	7 (5.3%)	0.69 (0.10–8.39)	0.55 (ns)
>20	1 (0.7%)	0.37 (0.03–2.22)		1 (0.8%)	1.46 (0.12–10.07)	
Serum AMH (ng/mL)						
≤4	9 (6.5%)	32.14 (4.70–355.3)	<0.001 *	6 (4.6%)	366 (25.05–4120)	<0.001*
> 4	28 (20.3%)	0.03 (0.00–0.21)		2 (1.5%)	0.00 (0.00–0.03)	

* p-value < 0.05 was considered significant. ns = non-significant. Abbreviations: AMH; anti-Müllerian hormone, FSH; follicle-stimulating hormone, LH; luteinizing hormone.

## Data Availability

The datasets used and/or analyzed during the current study are available from the corresponding author on reasonable request.

## List of Abbreviations

AMH	anti-Müllerian hormone
BMI	Body mass index
CC	Clomiphene Citrate
FSH	follicle-stimulating hormone
GRH	gonadotropin-releasing hormone
HCG	Human Chorionic Gonadotropin
ICSI	intracytoplasmic sperm injection
IVF	in vitro fertilization
IVM	in vitro maturation
LE	letrozole
LH	luteinizing hormone
MFG	Modified Ferriman–Gallwey scoring method
OHSS	ovarian hyper-stimulation syndrome
OI	Ovulation induction
OR	Odds Ratio
PCOS	Polycystic ovarian syndrome
TSH	thyroid-stimulating hormone
TVS	Transvaginal sonography
